# Binge Drinking and Occupation, North Dakota, 2004–2005

**Published:** 2007-09-15

**Authors:** Dwayne W Jarman, Timothy S Naimi, Stephen P Pickard, Walter W Randolph Daley, Anindya K De

**Affiliations:** Centers for Disease Control and Prevention (CDC), Epidemic Intelligence Service Officer assigned to the North Dakota Department of Health. Dr Jarman currently works with CDC as a Preventive Medicine Fellow; Division of Adult and Community Health, National Center for Chronic Disease Prevention and Health Promotion, CDC, Atlanta, Georgia; Division of Emergency Preparedness and Response, National Center for Public Health Informatics, CDC, CDC Career Field Officer, North Dakota Department of Health, Bismarck, North Dakota; Office of Workforce and Career Development, Career Development Division, CDC, Atlanta, Georgia; Office of Workforce and Career Development, Career Development Division, CDC, Atlanta, Georgia

## Abstract

**Introduction:**

Binge drinking is a leading cause of preventable death and results in employee absenteeism and lost productivity. Knowledge about the prevalence of binge drinking among employees of different occupations is limited.

**Methods:**

We assessed the prevalence of binge drinking (i.e., consuming five or more drinks per occasion during the previous 30 days) by primary occupation using data from the 2004–2005 North Dakota Behavioral Risk Factor Surveillance System. We used logistic regression to assess the association between binge drinking and primary occupation.

**Results:**

Overall, 24.1% (95% confidence interval [CI], 22.5–25.7) of North Dakota workers reported binge drinking. The prevalence was highest among farm or ranch employees (45.3%; 95% CI, 28.3–63.4), food or drink servers (33.4%; 95% CI, 23.9–44.4), and farm or ranch owners (32.5%; 95% CI, 26.3–39.4). The prevalence was lowest among health care workers (13.2%; 95% CI, 10.3–16.8). Compared with health care workers, the adjusted odds of binge drinking were highest among farm or ranch employees (adjusted odds ratio [AOR], 2.2; 95% CI, 0.9–5.5), food or drink servers (AOR, 2.1; 95% CI, 1.1–4.0), and farm or ranch owners (AOR, 1.7; 95% CI, 1.1–2.6). Health insurance coverage was lowest among employees in occupations with the highest prevalence of binge drinking.

**Conclusion:**

We found occupational differences in the prevalence of binge drinking among employees in North Dakota. Many occupational categories had a high prevalence of binge drinking. We recommend the implementation of both employer-sponsored and population-based interventions to reduce binge drinking among North Dakota workers, particularly because employees in occupations with the highest rates of binge drinking had the lowest rates of health insurance coverage.

## Introduction

Excessive drinking, including high per-occasion alcohol consumption (e.g., binge drinking) and high average daily alcohol consumption, is the third leading cause of preventable death in the United States (1). In 2001, excessive alcohol consumption accounted for 75,000 deaths (2). In 1998, the direct and indirect economic cost of excessive alcohol consumption was $185 billion (3). Binge drinking, defined as consuming five or more drinks on one or more occasions during the previous 30 days (4), is the most common type of excessive drinking and accounts for more than half of all alcohol-related deaths (2). Binge drinking is an important risk factor for unintentional injury, interpersonal violence, suicide, and adverse reproductive outcomes (2,5-11).

Work-related consequences of binge drinking include unintentional injuries, elevated health care costs, poor job performance, and absenteeism as a result of alcohol-induced hangover or other alcohol-related problems (12-16). Furthermore, lost productivity accounts for more than 70% of all costs attributable to excessive drinking (3). However, despite the substantial effects of binge drinking on employers and their employees, knowledge about the association between occupation and binge drinking is limited. Assessment of occupation-specific risk for binge drinking can provide information for guiding efforts to reduce binge drinking among workers (17-19).

North Dakota consistently has one of the highest rates of binge drinking in the nation (20). The purpose of this study was to assess rates of binge drinking and frequent binge drinking among occupational groups in North Dakota. Because occupation may determine health care coverage and therefore affect the availability of effective clinical interventions (e.g., brief counseling, intervention), we assessed health care access among North Dakota workers who reported binge drinking.

## Methods

The Behavioral Risk Factor Surveillance System (BRFSS) is an ongoing, state-based, random-digit–dialed telephone survey coordinated by the Centers for Disease Control and Prevention (CDC). The survey uses a disproportionate stratified sampling method and is conducted annually by all states (4). Information on health risk behaviors and preventive health practices related to the leading causes of death among the U.S. civilian, noninstitutionalized population aged 18 years or older is obtained from BRFSS data (21). Details of the BRFSS sampling methods, purpose, and method of analysis are published elsewhere (4,21,22). We conducted a population-based cross-sectional study of the association between binge drinking and primary occupation in North Dakota using 2004–2005 BRFSS data.

To our knowledge, North Dakota is the only state that collects information on occupation and binge drinking using the BRFSS. The BRFSS survey assesses employment status with the following question: "Are you currently: employed for wages, self-employed, out of work for more than 1 year, out of work for less than 1 year, a homemaker, a student, retired, or unable to work?" We defined people as "employed" if they reported their employment status as either "employed for wages" or "self-employed." Respondents not meeting criteria for being employed were defined as "not employed."

The North Dakota BRFSS began collecting information on occupation in 2004. Data on primary occupation were collected using the following question: "Which of the following most accurately describes the type of work or business you currently work in most often?" Occupational categories selected from the largest known employment categories in the state included state government employee, other government employee, farmer or rancher (i.e., farm or ranch owner), other farm or ranch worker (i.e., farm or ranch employee), manufacturing, health care, food or drink server (e.g., waiter, waitress, bartender), wholesale or retail sales, financial sales, and other. We defined "workers" as all employed respondents who selected one of nine occupation responses or the "other" occupation response. Employed respondents who did not provide their occupation were excluded from all occupation-related subanalyses.

We defined binge drinkers as adults who had consumed alcohol during the previous month and who answered "one" or a higher number to the following question: "Considering all types of alcoholic beverages, how many times during the past 30 days did you have five or more drinks on an occasion?" We defined frequent binge drinkers as those who reported binge drinking three or more times during the previous 30 days; we reported this number of frequent binge drinkers as a proportion of total binge drinking workers. Nonbinge drinkers were defined as either respondents who had not drunk alcohol during the previous 30 days (i.e., nondrinkers) or respondents who had drunk alcohol during the previous 30 days but who did not binge drink.

We also assessed health insurance coverage and usage among binge drinkers. We defined "having health care" as a yes response to the following question: "Do you have any kind of health care coverage, including health insurance, prepaid plans such as HMOs, or government plans such as Medicare?" Inability to access a doctor was defined as any respondent answering no to the following question: "Was there a time in the past 12 months when you needed to see a doctor but could not because of the cost?" (23).

We calculated both the crude and age group– and sex-standardized prevalence of binge drinking by occupation; we standardized the prevalence of binge drinking for age group and sex covariates to the North Dakota adult population aged 18 years or older to remove the effects of these factors on the prevalence of binge drinking by occupation in North Dakota (24). We used logistic regression to calculate the crude and adjusted odds of binge drinking by occupation; we used health care workers as the referent group. We controlled for sex, age group, marital status, annual income, and education as potential confounders when measuring the adjusted odds of binge drinking by occupation. We weighted the analysis to generalize results to the population of North Dakota. We conducted analyses using SAS callable SUDAAN version 9.0 (Research Triangle Institute, Research Triangle Park, North Carolina) to account for the complex sample design. We report crude measures unless otherwise noted.

## Results

A total of 7055 North Dakota adults aged 18 years or older participated in the BRFSS for 2004–2005. The total response rate was 62% in 2004 and 58% in 2005. On weighted analysis, 67.5% of North Dakotans were employed, and 93.8% of those who were employed provided information about their primary occupation. Of those who gave information on their occupation, 66.2% were classified into one of nine occupations, and the remaining 33.8% were classified as "other."

Overall, 19.8% of all North Dakota adults reported binge drinking on at least one occasion during the previous 30 days (Table 1). The prevalence of binge drinking among employed respondents was higher (24.1%) than the prevalence among nonemployed respondents (10.8%) (odds ratio [OR], 2.6; 95% confidence interval [CI], 2.1–3.3). The odds of binge drinking remained higher among the employed respondents even after adjusting for age group and sex (OR, 1.6; 95% CI, 1.3–2.0) (data not shown). Compared with nonemployed respondents, employed respondents had a higher prevalence of binge drinking in every stratum of each variable assessed in the study (Table 1).

**Figure 1:**
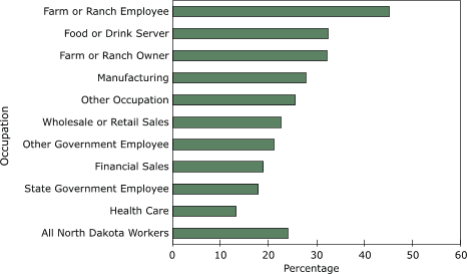
Prevalence of binge drinking by occupation, North Dakota, 2004–2005. Binge drinking was defined as having consumed five or more drinks on one or more occasions during the previous 30 days.

Among employed respondents, the prevalence of binge drinking varied by occupation, ranging from 13.2% among health care workers to 45.3% among farm or ranch employees (Table 2, Figure 1). Overall, the prevalence of binge drinking exceeded 20% among 7 of the 10 occupational categories, and exceeded 25% in 5 of the 10. The prevalence of binge drinking exceeded 30% for farm or ranch employees, food or drink servers, and farm or ranch owners, which comprise approximately 14% of the employed people in North Dakota. After standardizing by age group and sex, the prevalence of binge drinking remained the highest among farm or ranch employees and lowest among health care workers.

**Table:** 

Farm or ranch employees, food or drink servers, farm or ranch owners, manufacturing employees, employees who selected "other" occupation, wholesale or retail sales employees, and other government employees had significantly higher odds of binge drinking compared with health care workers (Table 3). We found that marital status, age group, and sex accounted for most of the difference in the crude and adjusted odds among participants in many occupations. Even after adjusting for multiple potential confounders, the odds of binge drinking remained significantly higher among food or drink servers, farm or ranch owners, and among people employed in the "other" occupation category compared with health care workers.

**Figure 2:**
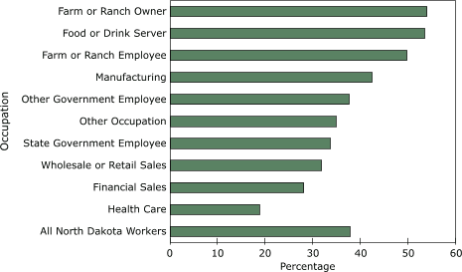
Prevalence of frequent binge drinking by occupation among workers who reported binge drinking, North Dakota, 2004–2005. Binge drinking was defined as having consumed five or more drinks on one or more occasions during the previous 30 days. Frequent binge drinking was defined as binge drinking on three or more occasions during the previous 30 days.

More than one-third (37.6%; 95% CI, 33.7–41.6) of binge drinking workers reported frequent binge drinking (three or more binge drinking occasions during the past 30 days) (data not shown). Among binge drinkers, the prevalence of frequent binge drinking was generally more common among participants in occupations that also had a high prevalence of binge drinking (Figure 2). For example, half of all binge-drinking farm or ranch owners, their employees, and food or drink servers (the three occupations with the highest prevalence of binge drinking) were frequent binge drinkers.

**Table:** 

Among workers who reported binge drinking, 81.3% had some form of health care coverage (Table 4), and occupational groups with the lowest levels of coverage were the most likely to binge drink (Table 3). Although only 8.4% of workers who binge drink reported cost as a barrier to seeking medical care, workers who binge drink and who lack health insurance were more likely to report cost as a barrier to health care than were workers with health care coverage (26.6% vs 4.2%) (data not shown). Among workers who binge drink, similar proportions of frequent (three or more binge drinking occasions per month) and nonfrequent binge drinkers reported having health care coverage (75.9% vs 84.5%) and that cost was a barrier to seeking medical care (10.0% vs 7.4%) (data not shown).

## Discussion

Excessive alcohol consumption, including binge drinking, has enormous implications for business and the economy. Lost productivity accounts for approximately three-quarters of the costs of excessive drinking in the United States (3). In this study, we found that approximately one-quarter of all employed people in North Dakota reported binge drinking at least once in the past month, and that employed people were more likely to report binge drinking than those who were nonemployed, even after adjusting for age and sex. One-third of employed people who binge drink reported frequent binge drinking. Furthermore, people employed in occupations with the highest adjusted odds of binge drinking also had the lowest rates of health care coverage.

To our knowledge, this is the first population-based study to examine the relationship between binge drinking and occupation. Although no other study has focused on binge drinking, other studies support our findings that people in certain occupational groups (e.g., food or drink servers, agricultural workers) have higher rates of alcohol-related diagnoses compared with people in other occupational groups (e.g., health care workers) (25-27). Further work should be done to confirm whether these findings are similar in other states or nationally and to examine binge drinking rates for people in other occupations that we were unable to assess.

Although we did not establish a temporal relationship between work and binge drinking, binge drinking among workers can negatively affect the employer, regardless of when the binge drinking occurs. Binge drinking is associated with adverse occupational outcomes (7,12,28), and the presence and frequency of binge drinking is a strong predictor of occupational or industrial-related injuries (14). In addition, medical- and lost productivity–related costs are incurred by employers, regardless of the fact that most alcohol consumption occurs outside typical working hours (27).

It was impossible to establish a causal association between occupation and binge drinking during this cross-sectional study. Furthermore, even after controlling for established risk factors for binge drinking, some occupation-specific differences in binge drinking are probably due to characteristics of employees in certain occupations rather than the occupations themselves. Examples of key factors that we could not assess included familial country of origin, religious affiliation, history of alcohol use before employment, and coworker attitudes toward risk-taking behaviors (29).

Employment in certain occupations may be a risk factor for binge drinking. Mandell et al found a cause-and-effect relationship between occupation and alcohol dependence (26), which might indicate a causal relationship between occupation and binge drinking. For example, workers employed in settings where alcohol is sold typically have easy access to alcohol and might work in social climates that are accepting of excessive drinking. In addition, workers who are self-employed or whose jobs are socially isolating might be at risk for excessive drinking as a result of a lack of peer feedback that discourages excessive drinking or drinking on the job. Additionally, during off-season times when less work is available among workers employed in seasonal occupations (i.e., winter for farm or ranch owners and employees), boredom and inactivity might contribute to binge drinking.

This study has some limitations. Our prevalence estimates of binge drinking are probably conservative, because alcohol use is typically underreported by respondents (30) and because nonrespondents may drink more excessively than survey respondents. However, whether underreporting differs between employed respondents and nonemployed respondents or if certain occupational groups might be more likely to underreport compared with other groups is unknown (26). Furthermore, because our sample size required us to combine certain occupations into relatively broad categories and because other occupations are not common in North Dakota and therefore not represented among North Dakota BRFSS respondents, we might have omitted occupations with a high prevalence of binge drinking. Finally, our sample and conclusions are restricted to North Dakota; an analysis of data collected in other states might have produced different results.

We recommend the aggressive implementation of effective population-based policy interventions to reduce excessive drinking, because binge drinking was common among most occupational groups and because a substantial proportion of workers in some occupations with particularly high rates of binge drinking were most likely to lack health insurance or report cost as a barrier to health care. Examples of effective policy interventions include increasing alcohol excise taxes, limiting the density of alcohol outlets and hours of sale, and enforcing laws prohibiting the sale of alcohol to people already intoxicated (31-35). Employer-based programs (e.g., employee assistance programs, employee wellness programs) are additional strategies that can be effective in reducing binge drinking among employees (17,18). However, self-employed workers or workers in small businesses often do not have such programs available to them.

Although many employed people might lack access to worksite health programs, the majority of workers in this analysis reported having health insurance coverage, and only one-tenth of workers reported that cost was a barrier to accessing health care. The U.S. Preventive Services Task Force recommends routine screening and brief counseling interventions (SBI) in primary-care settings for alcohol "misuse" (i.e., excessive drinking) (36). In controlled trials, SBI typically reduces total alcohol consumption by 20% and also reduces the number of binge-drinking episodes (36,37). However, despite the fact that a recent report by the Partnership for Prevention determined that SBI for alcohol misuse is one of the most valuable of the recommended clinical preventive services, SBI is one of the least commonly performed of these services, and less than 20% of employer-sponsored health plans cover SBI (38). The National Business Group on Health, a coalition of large businesses that purchase health care coverage for their employees, recently called for parity in coverage between physical problems (e.g., diabetes) and mental health and substance abuse problems (39). Efforts to work with businesses to negotiate SBI coverage when purchasing health insurance for their employees will be another important way to help prevent and reduce binge drinking among employed people.

## Figures and Tables

**Table 1:** Prevalence of Binge Drinking^a^ Among North Dakota Adults, by Selected Characteristics, 2004–2005

Characteristic	Employed^b^,^c^		Nonemployed^c^,^d^		All Adults^c^	

	Population Estimate, N^e^	Binge Drinking Prevalence, % (95% CI)	Population Estimate, N^e^	Binge Drinking Prevalence, % (95% CI)	Population Estimate, N^e^	Binge Drinking Prevalence, % (95% CI)
**Age group, y**						
18-20	13,019	28.8 (18.5-41.8)	14,614	24.4 (15.5-36.2)	27,632	26.5 (19.4-35.0)
21-35	105,830	36.0 (32.7-39.5)	27,423	31.9 (25.0-39.8)	133,252	35.2 (32.1-38.4)
36-49	108,104	22.2 (20.1-24.5)	13,221	9.5 (5.9-14.9)	121,325	20.8 (18.9-23.0)
≥50	98,336	12.7 (11.1-14.5)	99,331	3.2 (2.4-4.3)	197,667	7.9 (7.0-9.0)
**Sex**						
Male	179,701	32.9 (30.6-35.4)	59,167	17.2 (13.6-21.5)	238,868	29.1 (27.0-31.1)
Female	146,623	13.2 (11.6-14.9)	96,898	6.9 (5.2-9.0)	243,521	10.6 (9.5-12.0)
**Race**						
White	311,491	24.1 (22.6-25.8)	142,102	10.6 (8.7-12.7)	453,593	19.9 (18.7-21.2)
American Indian/ Alaskan Native	8,697	28.4 (19.4-39.5)	10,005	17.8 (9.7-30.3)	18,702	22.7 (16.2-30.9)
Other	5,414	14.3 (5.3-33.3)	2,711	1.2 (0.2-8.3)	8,125	10.0 (3.8-23.7)
**Marital Status**						
Married	222,555	19.2 (17.7-20.8)	83,879	6.2 (4.8-8.1)	306,433	15.6 (14.4-16.9)
Divorced	24,839	26.1 (22.0-30.6)	9,050	10.8 (6.2-18.2)	33,888	22.0 (18.6-25.8)
Widowed	7,021	5.1 (2.6-10.0)	28,051	1.7 (0.7-3.7)	35,072	2.4 (1.4-4.0)
Separated	2,112	20.9 (10.3-37.6)	794	14.5 (3.5-44.1)	2,906	19.1 (10.3-32.9)
Never married	58,540	38.6 (33.7-43.9)	31,143	28.1 (21.4-35.9)	89,683	35.0 (30.9-39.3)
Member of an unmarried couple	10,370	54.4 (43.2-65.2)	2,745	47.0 (25.8-69.2)	13,115	52.9 (42.8-62.7)
**Income, $**						
<25,000	54,699	27.0 (22.8-31.6)	56,971	12.6 (9.5-16.4)	111,670	19.6 (17.0-22.6)
25,000-49,999	114,363	27.0 (24.4-29.9)	42,601	8.1 (5.6-11.4)	156,964	21.9 (19.8-24.2)
≥50,000	128,645	22.5 (20.3-24.8)	23,650	11.6 (8.0-16.5)	152,295	20.8 (18.8-22.9)
**Education**						
Less than college	104,662	27.2 (24.4-30.3)	75,513	6.8 (5.0-9.3)	180,175	18.7 (16.7-20.8)
Some or more college	221,415	22.6 (20.8-24.4)	80,119	14.6 (11.7-17.9)	301,534	20.4 (18.9-22.0)
**Employment Status**						
Employed for wages	267,110	23.5 (21.9-25.3)	0	NA	267,110	23.5 (21.9-25.3)
Self-employed	59,213	26.4 (22.8-30.5)	0	NA	59,213	26.4 (22.8-30.5)
Out of work (<1 year)	0	NA	4,296	13.7 (4.5-34.9)	4,296	13.7 (4.5-34.9)
Out of work (>1 year)	0	NA	8,097	19.1 (11.1-31.0)	8,097	19.1 (11.1-31.0)
Homemaker	0	NA	29,884	4.5 (2.8-7.2)	29,884	4.5 (2.8-7.2)
Student	0	NA	28,350	34.7 (27.2-43.1)	28,350	34.7 (27.2-43.1)
Retired	0	NA	72,532	3.5 (2.5-4.8)	72,532	3.5 (2.5-4.8)
Unable to work	0	NA	11,417	7.2 (3.7-13.7)	11,417	7.2 (3.7-13.7)
Total	326,323	24.1 (22.5-25.7)	156,065	10.8 (9.0-12.9)	482,388	19.8 (18.6-21.0)

CI indicates confidence interval; NA, not applicable.

a Binge drinking was defined as having consumed five or more drinks on one or more occasions during the previous 30 days.

b Employed is defined as working for wages or being self-employed.

c Represents prevalence of binge drinkers by group with the selected characteristic; therefore, percentages do not total 100.

d Nonemployed is defined as one of the following: out of work for more than 1 year, out of work for less than 1 year, a homemaker, a student, retired, or unable to work.

e Indicates the total weighted population estimate of all North Dakota adults with the selected characteristic.

**Table 2:** Prevalence of Binge Drinking^a^ Among North Dakota Workers^b^, by Occupational Category, 2004–2005

Occupational Category	Population Estimate, N^c^	Proportion of Workers, % (95% CI)^d^	Prevalence of Binge Drinking, % (95% CI)^d^	Standardized Prevalence^e^ of Binge Drinking, % (95% CI)^d^
Farm or ranch employee	3,484	1.1 (0.8-1.6)	45.3 (28.3-63.4)	37.1 (28.8-46.3)
Food or drink server	12,208	4.0 (3.2-4.9)	33.4 (23.9-44.4)	28.5 (19.6-39.5)
Farm or ranch owner	26,381	8.6 (7.6-9.6)	32.5 (26.3-39.4)	23.7 (19.2-28.9)
Manufacturing	26,033	8.4 (7.4-9.6)	28.0 (22.1-34.8)	22.5 (17.6-28.3)
Other occupation	104,230	33.8 (32.2-35.4)	26.4 (23.7-29.3)	23.9 (21.5-26.5)
Wholesale or retail sales	30,431	9.9 (8.9-10.9)	23.8 (19.4-28.8)	21.3 (17.4-25.8)
Other government employee	26,317	8.5 (7.6-9.5)	21.5 (16.6-27.3)	17.2 (13.6-21.6)
Financial sales	12,340	4.0 (3.4-4.7)	18.6 (12.8-26.2)	20.5 (13.9-29.0)
State government employee	27,965	9.1 (8.2-10.1)	17.6 (13.6-22.4)	16.5 (12.8-21.1)
Health care	39,148	12.7 (11.7-13.8)	13.2 (10.3-16.8)	14.4 (10.9-18.7)

CI indicates confidence interval.

a Binge drinking was defined as having consumed five or more drinks on one or more occasions during the previous 30 days.

b Workers was defined as all respondents employed in one of the nine occupational categories or the "other" occupation category.

c Indicates the total weighted population estimate of all North Dakota adults employed in the selected occupational category.

d Represents the percentage of occupation; therefore, percentages do not total 100.

e Prevalence standardized by age group (18–20 years, 21–35 years, 36–49 years, or ≥50 years) and sex to the North Dakota adult population age ≥18 years. Standardization removes the effects of these factors on the prevalence of binge drinking by occupation.

**Table 3:** Crude and Adjusted Odds of Binge Drinking^a^ Among North Dakota Workers^b^, by Occupational Category, 2004–2005

Occupational Category	Crude OR (95% CI)	AOR^c^ (95% CI)
Farm or ranch employee	5.4 (2.5-12.0)	2.2 (0.9-5.5)
Food or drink server	3.3 (1.9-5.7)	2.1 (1.1-4.0)
Farm or ranch owner	3.2 (2.1-4.8)	1.7 (1.1-2.6)
Manufacturing	2.6 (1.7-3.9)	1.2 (0.8-1.9)
Other occupation	2.4 (1.7-3.2)	1.5 (1.1-2.0)
Wholesale or retail sales	2.1 (1.4-3)	1.5 (1.0-2.3)
Other government employee	1.8 (1.2-2.7)	1.1 (0.7-1.7)
Financial sales	1.5 (0.9-2.5)	1.4 (0.8-2.6)
State government employee	1.4 (0.9-2.1)	1.1 (0.7-1.7)
Health care	1.0 (Ref)	1.0 (Ref)

CI indicates confidence interval; OR, odds ratio; AOR, adjusted odds ratio; ref, reference group.

a Binge drinking was defined as having consumed five or more drinks on one or more occasions during the previous 30 days.

b Workers was defined as all respondents employed in one of the nine occupational categories or the "other" occupation category.

c Odds ratio adjusted for marital status (married, divorced, widowed, separated, never married, member of an unmarried couple), sex, age group (18–20 years, 21–35 years, 36–49 years, or ≥50 years), annual income (<$25,000, $25,000–$49,999, ≥$50,000), and education (less than college, some or more college).

**Table 4:** Prevalence^a^ of Having Some Type of Health Care Coverage and No Cost Barrier to Doctor Visits Among Workers Who Binge Drink^b^, by Occupational Category, North Dakota, 2004–2005

Occupational Category	Have Some Type of Health Care Coverage^c^, % (95% CI)	No Cost Barrier to Doctor Visits^d^, % (95% CI)
All North Dakota workers	81.3 (77.5-84.6)	91.6 (89.1-93.6)
Other government employee	95.3 (79.2-99.1)	97.2 (82.9-99.6)
State government employee	94.7 (86.0-98.1)	94.2 (82.8-98.2)
Health care	93.3 (84.1-97.3)	92.2 (82.7-96.7)
Financial sales	92.8 (70.5-98.6)	100 (NA)
Wholesale or retail sales	90.9 (82.7-95.4)	93.3 (85.6-97)
Manufacturing	86.9 (74.2-93.9)	88.0 (74.1-95)
Farm or ranch employee	81.6 (58.8-93.2)	100 (NA)
Other occupation	81.0 (74.5-86.2)	92.4 (88.1-95.2)
Farm or ranch owner	60.6 (46.0-73.5)	93.7 (86.0-97.3)
Food or drink server	40.9 (24.0-60.3)	66.6 (47.4-81.6)

CI indicates confidence interval; NA, not applicable.

a Represents prevalence of binge drinkers by occupation; therefore, percentages do not total 100.

b Binge drinking was defined as having consumed five or more drinks on one or more occasions during the previous 30 days.

c Data on health care coverage were collected using the following question: "Do you have any kind of health care coverage, including health insurance, prepaid plans such as HMOs, or government plans such as Medicare?"

d Data on no cost barrier to doctor visits were collected using the following question: "Was there a time in the past 12 months when you needed to see a doctor but could not because of the cost?"
